# Mushroom Bioactive Molecules as Anticancerous Agents: An Overview

**DOI:** 10.1002/fsn3.70580

**Published:** 2025-07-14

**Authors:** Ali Ikram, Nasir A. Ibrahim, Muhammad Tayyab Arshad, Abroo Fatima, Ali Asghar Taseer, Mahreen Faqeer Hussain, Zunair Abdullah, Nosiba S. Basher, Muhammed Adem Abdullahi, Ammar AL‐Farga, Mohammed Ali Al‐Duais

**Affiliations:** ^1^ University Institute of Food Science and Technology The University of Lahore Lahore Pakistan; ^2^ Department of Biology, College of Sciences Imam Mohammad Ibn Saud Islamic University (IMSIU) Riyadh Saudi Arabia; ^3^ Functional Food and Nutrition Program, Faculty of Agro‐Industry Prince of Songkla University Hatyai, Songkhla Thailand; ^4^ Department of Gynecology and Obstetric Aziz Fatima Hospital Faisalabad Faisalabad Pakistan; ^5^ Department of Pediatrics Faisalabad Medical University, Allied Hospital Faisalabad Pakistan; ^6^ University Institute of Diet and Nutritional Sciences The University of Lahore Lahore Pakistan; ^7^ Department of Food Science and Postharvest Technology, Jimma University College of Agriculture and Veterinary Medicine Jimma University Jimma Ethiopia; ^8^ Biochemistry in Department of Biological Sciences, College of Science University of Jeddah Jeddah Saudi Arabia; ^9^ Department of Biochemistry, Faculty of Science University of Tabuk Tabuk Saudi Arabia

**Keywords:** cancer, leukemia, mushroom, tumor

## Abstract

Mushrooms have long been used in Traditional Chinese medicine (TCM), where they play an important role in promoting overall health and well‐being. However, the therapeutic benefits of mushrooms have made this group of macrofungi a significant part of traditional medicine, particularly in Southeast Asia and China. Across the globe, cancer is the leading cause of death. Powerful anti‐cancer medications known as traditional chemotherapeutic agents treat this dangerous disease. However, patients are always accompanied by immunosuppression, increasing the risk of tumor return and mortality. Identifying, separating, and transferring bioactive macromolecules naturally present in tumor‐genic foods could be a promising option. Mushrooms are a source of macromolecules such as ergosterol, p‐hydroxybenzoic acid, linoleic acid, β‐glucan, α‐glucan, resveratrol, concanavalin A, Cibacron blue affinity protein, and others. Numerous studies have demonstrated that oyster mushroom extracts are full of macromolecules like β‐glucan and other polysaccharides that inhibit the proliferation of cancer cell types without affecting healthy cells. The genera *Phellinus, Pleurotus, Agaricus, Ganoderma, Clitocybe, Antrodia, Trametes, Cordyceps, Xerocomus, Calvatia, Schizophyllum, Flammulina, Suillus, Inonotus, Inocybe, Funlia, Lactarius, Albatrellus, Russula, and Fomes* are the mushrooms that have been linked to success against cancer. The anticancerous substances are essential because they create reactive oxygen species, inhibit mitotic kinase, prevent mitosis, inhibit angiogenesis, and topoisomerase, which ultimately stop cancer growth. This review provides the most recent results on the pharmacologically active chemicals, their potential as antitumor agents, and the underlying mechanism of biological activity.

## Graphical abstract

This review discusses the molecular mechanisms through which these compounds inhibit cancer progression, including the induction of reactive oxygen species, inhibition of mitotic kinase and angiogenesis, and suppression of topoisomerase activity. By providing the latest insights on these anti‐tumor agents and their pharmacological actions, our review aims to contribute valuable information to the field of cancer research and support the development of novel therapeutic strategies.

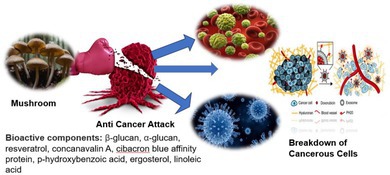

## 1 Introduction

In terms of morbidity and death, cancer is the world's number one public health issue. Some of the most prevalent cancer treatment choices include chemotherapy, radiation therapy, and surgical excision of tumors. However, these conventional methods come with several harmful side effects. One sign of therapy‐related side effects in cancer patients is anorexia (Nowakowski et al. 2021). Other symptoms include paresthesia, nausea and vomiting, exhaustion, and persistent pain. To avoid the drawbacks of presently prescribed medications, health sciences researchers have concentrated chiefly on medicinal plants or natural compounds with anti‐cancer capabilities (Bertollo et al. 2022).

Clinical trial‐based research has shown that herbal treatments, whether taken alone or combined with conventional therapy, positively benefit cancer patients' immunological control, survival, and quality of life. Consequently, combining plant‐based drugs with different mechanisms may synergistically increase the therapeutic efficacy of the therapies (Ju et al. 2022). Public health is seriously threatened by cancer in both high‐ and low‐income nations. GLOBOCAN estimates that 9.6 million people passed away due to cancer globally in the year 2018, making it the second most significant cause of death after cardiovascular illnesses (Raisi‐Estabragh et al. 2023).

A healthy diet and other modifiable risk factors are thought to be important in the prevention of cancer. Because of their distinct flavor and significance in a healthy diet low in calories, carbs, salt, fats, and cholesterol (Collatuzzo and Boffetta 2023), mushrooms have been used as a helpful meal by numerous civilizations for ages (Badalyan and Zambonelli 2023). Phyto‐chemical substances (alkaloids, phenolic acids, alkaloids, carotenoids and flavonoids), fiber, polysaccharides, vitamins (such as thiamin, niacin, ascorbic acid, riboflavin, and vitamins B and D), selenium, as well as the essential antioxidants ergothioneine and glutathione, which may play a substantial part in preventing cancer are also abundant in edible mushrooms (Arshad et al. 2025; Łysakowska et al. 2023).

The unique antioxidant ergothioneine is expected to play a key role in a number of the benefits of mushrooms, which are assumed to be conciliated by their characteristics as antioxidants (Liuzzi et al. 2023). Mushroom is an effective food that offers advantages for well‐being and the conventional nutrients it contains. However, understanding of culinary mushrooms composition and nutritional worth remained restricted till the previous 10 years compared to the species of medicinal mushrooms and vegetables. Because gourmet mushrooms have been considered a delicacy and have only been consumed seldom in many industrialized nations, they have received minimal attention from researchers (De Cianni et al. 2023).

Since the beginning of civilization, people have employed edible mushrooms to enhance flavor and meet nutritional demands. Proteins, polysaccharides, β‐glucan, ergosterol, levostatin, and triterpenes are among the pharmacologically significant bioactive macromolecules that are found to be abundant in it and can provide it anti‐neoplastic characteristics (Mishra et al. 2021). Mushrooms have a variety of health benefits, including anticancer, antioxidant, antiallergic, antidiabetic, cardiovascular protector, immuno‐modulatory, anticholesterolemic, antibacterial, antiviral, antifungal, antiparasitic, detoxification, as well as hepatic protective effects. These food substances have antitumor and anti‐inflammatory properties (Rani et al. 2023).

In the past 10 years, the interest in mushrooms' potential as pharmaceuticals has grown quickly. It has even been hypothesized that many of the mushrooms serve as miniature factories in pharmaceutical fields, creating substances consisting of exceptional biological capabilities. Additionally, novel medications against aberrant molecular and biochemical signals that cause cancer can now be developed thanks to the increased understanding of the molecular basis of carcinogenesis and metastasis (Sisodiya et al. 2023).

Various biomolecules with nutritional and therapeutic characteristics may be found in wild and domesticated mushrooms. Due to their nutritional and medicinal qualities, they have gained recognition as functional foods and as a source for creating medications and nutraceuticals (Semwal et al. 2023).

*Pleurotus ostreatus* (Jacq.ex.fr) P. kumm is a binomial name. About 40 unique species of the *Pleurotus* genus are known as “oyster mushrooms” and are extensively distributed in subtropical and tropical regions. They are very simple to artificially culture: *P. eryngii*, *P. sapidus*, *P. ulmarium*, *P. tuberegium*, *P. pulmonarius*, *P. geesteranus*, *P. citrinopileatus*, *Lentinus sajor‐caju*, *P. ostreatus*, *P. florida*, *P. highbing*, *P. flabellatus*, and *P. cystidiosus*; also, more species are included in the *Pleurotus* genus (Mishra et al. 2021).

The scientific and colloquial names both allude to the fruiting body's form. While the English common name oyster and the Latin *ostreatus* allude toward the form of the cap that has a resemblance to the bi‐valve of the identical term, *Pleurotus* (sideways) mentions the sideways development of the stem part regarding the cap (Ghosal 2023). About 100 distinct bioactive substances may be found in the *P. ostreatus* fruiting body, primarily considered a possible recent origin of dietary fiber (Zhao et al. 2024).

Fungal cell walls are abundant in non‐starchy polysaccharides comparatively, of which beta‐glucan is the most engaging working component. Additionally, they contain phenolic compounds such as protocatechuic acid, gallic acid, homogentisic acid, chrysin, rutin, myricetin, and naringin, as well as tocopherols like ascorbic acid and tocopherol (Ishara et al. 2022). They are also nutritious meals that are higher in carbs, lipids, protein, vitamins, and minerals but lower in fat and calories (Jurkaninová et al. 2024).

Another name for it is button mushroom *Agaricus bisporus*, which contains numerous significant bioactive chemicals and is a good food source. The bioactive ingredients have nutritional benefits (Batool et al. 2023). The benefits of *A. bisporus* on human health are numerous. Numerous studies have also discussed the significance of this mushroom in the cosmetics sector due to specific ingredients that improve facial attractiveness by addressing various skin issues (Usman et al. 2021).

The mushrooms were historically used alone or in combination as herbal therapeutics in Asian countries. To this day, we are familiar with the fact that a variety of mushrooms have the notable capacity to aid our bodies in recovering from a few of the significant harmful and complicated diseases, including AIDS, cancer, cardiovascular diseases, and diabetes, along with many other health issues (Hamza et al. 2023).

Mushroom is thought to be a viable source of medication currently. Antifungal, antibacterial, antiprotozoal, anticancer, and antiviral activities of the mushrooms have been observed. During the season of humidity from March to September, wild mushrooms are typically picked from forested areas. Since the invention of the plastic bag technique in 1990, it has become simple to grow edible and therapeutic mushrooms year‐round on a variety of agricultural wastes. In many nations, mushroom production is a tiny but rapidly expanding business (Osuafor et al. 2023).

Approximately 14,000 species have been identified recently, 2000 of which are suitable for human consumption. However, 30–50 different species of mushrooms are now grown in about 100 countries worldwide and are regarded as one of the best and most affordable food sources (Khan et al. 2024). Some of the kinds that are being utilized on commercial levels in the world include *Auricular* spp. *and Coronus comates, Agaricus bisporus, Lentinus edodes, Flammulina velutipes, Phellorinia inquinans, Stropharia rugosoannulata, Pleurotus ostreatus*, and *Volvariella volvacea* (Rehman et al. 2024).

*P. ostreatus* was examined for its anticancer effects against human PC‐3 cells for androgen‐independent prostate cancer (Pandey et al. 2023). Additionally, it was shown that a water‐soluble source primed from the newly acquired *P. ostreatus* caused the greatest addition to cytotoxicity, dose‐dependent promoted apoptosis in PC‐3 cells (Meng, Jang, et al. 2023; Meng, Niu, et al. 2023). An extract from the liquid polysaccharide edible mushroom, *P. ostreatus*, is thought to have pro‐apoptotic and antiproliferative actions against HT29 colon cancer cells (Rangsinth et al. 2023).

Due to the existence of a recently discovered glucan with a low molecular weight that has encouraging antitumor‐genetic properties and has been shown to directly impact cell proliferation in colon cancer by inducing mechanical cell death (Sun et al. 2025; Tsubaki et al. 2023). Additionally, *P. ostreatus* hot water essence prevented MCF‐7 breast cancer cells from growing in humans. *P. ostreatus* methanol extract had an effect on a few breast and colon cancer cells. The extract prevented the proliferation of healthy colon FHC cells and epithelial mammary cells, while it subdued breast and colon cancer cell development (Fordjour et al. 2023).

## 2 Nutritional Components of Mushroom

On a dry weight base, the maturing bodies of mushrooms hold 50%–65% of their entire weight in carbohydrates. It is made up of oligosaccharides, monosaccharides, and their derivatives. The sugars in the carbs, such as mannitol and trehalose, are somewhat alcoholic. When subjected to environmental conditions, including cold, heat, desiccation, oxidation, etc., trehalose is known to manufacture stress‐responsive factors in human cells while maintaining cellular integrity (Hassan et al. 2023).

Avoiding protein denaturation, which typically degrades under stressful circumstances, may be the mechanism at work here. All necessary amino acids are present in mushrooms high in protein, and glutamic acid, aspartic acid, and arginine are three of these abundant amino acids (Farhan and Chechan 2023). The two unique amino acids ornithine, recognized for its odd physiological actions, and g‐amino butyric acid (GABA), a non‐essential amino acid, have also been discovered. Unsaturated fatty acids may be found in mushrooms with a total lipid (crude fat) composition of 20–30 g/kg DM. They are abundant in linoleic and oleic acids (Östbring et al. 2023).

In animal models of the prostate, colon, and breast cancers, linoleic acid has been shown to have anticarcinogenic effects on nearly all stages of tumorigenesis. It has also been shown to slow the growth of tumor cells. Additionally, 1‐octen‐3‐ol, a class of alcoholic mushrooms that is a major fragrant component in mushroom flavor, is a linoleic acid precursor. Tocopherol, a crucial antioxidant component, is present in the lipid portion of mushrooms. Particularly rich in vitamin B complex and vitamin D, mushrooms are a wonderful source of vitamins (Delcros et al. 2023).

Interestingly, mushrooms are the only plant foods containing vitamin D. Recent research has shown that mushrooms create quantities of vitamin D2 significantly greater than the daily needs when exposed to UV radiation under specific circumstances (Neill et al. 2023). Producing vitamin D2 involves a photochemical reaction that occurs at a rate higher than required to meet daily needs. Ergosterol, a sterol found in fungi, is transformed into vitamin D2 through a sequence of photochemical and thermal reactions accelerated by ultraviolet (UV) radiation from daylight. Potassium, calcium, phosphorus, and magnesium are abundant in mushrooms (Shrestha et al. 2023).

Moreover, mushrooms have a relatively low sodium content compared to other vegetables, and they are considered a healthy alternative for those with hypertension. Fresh mushrooms have been shown to reduce total cholesterol levels, which is excellent for controlling cardiovascular disorders (Uffelman et al. 2023). They also include soluble and non‐soluble fibers. Humans benefit greatly from the existence of dietary fiber (DF) and non‐dietary carbohydrates (NDCs), such as polysaccharide–protein complexes (PSPC), b‐glucans, xylans, chitin, mannans, hemicelluloses, and galactose. The main ones include 3‐octanol, 3‐octone, 1‐octen‐3‐ol, benzaldehyde, 2‐octen‐1‐ol, and octanol. Mushrooms also contain volatile chemicals integrated with certain enzyme systems adept at catabolizing fragrant substrates (An et al. 2023).

## 3 Types of Cancers and Their Main Reasons

### 3.1 Thyroid Cancer

Thyroid carcinoma (TC) is one of the most prevalent endocrine malignancies, making up 3.4% of all malignancies (Paladino et al. 2023). The most widely recognized explanation of follicular cell carcinogenesis states that thyroid follicular cells undergo a multistep process that may end in differentiated or undifferentiated TC. In this paradigm, unique molecular abnormalities at some distinct phases drive the development of poorly differentiated to well‐established thyroid cancer. The notion of cancer‐stem‐like cells, which states that a small subset of stem cells may produce cancer cells with varied phenotypes after undergoing genetic and epigenetic changes, has been introduced (Davies et al. 2023).

Among the thyroid carcinomas, over 90% of thyroid cancers are well‐differentiated, with papillary thyroid cancer (PTC) and follicular thyroid cancer (FTC) making up the majority (FTC) (Luvhengo et al. 2023). Anaplastic thyroid cancers (ATC) and undifferentiated thyroid cancers are uncommon cancers, about 1% and 5%, respectively, characterized by aggressive activity and a relatively brief average survival period of 5 years and 6 months (Gu et al. 2024).

Parafollicular C cell cancers, known as medullary thyroid cancers (MTC), account for the remaining 5% of thyroid carcinoma cases. The availability of the genome sequence during the past 30 years has dramatically advanced our understanding of the molecular processes driving TC. All tumors in TC have a very low load of somatic mutations, making it a genetically straightforward illness. More than 90% of TC cases include driver mutations, which are alterations that assist the selective growth of cancer cells (Bratic Hench et al. 2023).

The deregulation of two pathways causes significant TC: phosphatidylinositol‐3 kinase (PI3K)/AKT and mitogen‐activated protein kinase (MAPK) signaling pathways. The MAPK pathway is activated through mutations involving TRK and RET/PTC; MAPK activation is a prerequisite for PTC initiation. While triggering mutations in RAS, PIK3CA, and AKT1, in addition to the inactivation of PTEN, which causes downregulation of this pathway, can lead to PI3K/AKT activation, which is considered crucial in the onset of FTC. Additional mutations impacting other cell signaling pathways, such as p53 and Wnt/catenin, are involved in TC development and dedifferentiation to PDTC and ATC. Lately, mutations of TERT activator have been identified in the histology of all TC, with a more significant occurrence in aggressive and poorly differentiated tumors, demonstrating their relevance in TC development. The majority of MTC cases develop by specific abnormalities of RET (Rearranged during transfection) proto‐oncogene, which are sporadic/hereditary germline processes in several endocrine neoplasia type 2A (MEN2A) and 2B (MEN2B) pathologies. Only a tiny proportion of sporadic MTC is caused by mutations in H‐, K‐, and N‐RAS (Paladino et al. 2023).

### 3.2 Prostate Cancer

As for the sixth most common cause of death, prostate cancer is the second most common type of cancer in terms of frequency of diagnosis and incidence in men worldwide, with 1,414,259 new cases and 375,304 related deaths anticipated to have occurred globally in 2020 (Sung et al. 2021).

The rate of incidence of prostate cancer differs remarkably by region, with Asia historically having the lowest rates (Jiang et al. 2024). However, the cases of prostate cancer are rising significantly as a result of economic growth, an increase in life expectancy, and Western culture (Jalloh et al. 2024). In 20 out of 47 Asian nations in 2020, prostate cancer was reported to be among the three most frequent malignancies in males. It was the most frequent in Lebanon, Israel, Kuwait, Qatar, Oman, Japan, and the United Arab Emirates (Chiong et al. 2024).

In contrast to this tendency, the incidence in Western countries has either plateaued or is dropping (Culp et al. 2020). One of the primary therapeutic targets for prostate cancer has been identified as inhibiting the androgen receptor (AR), which plays a major role in the development of prostate cancer (Lam et al. 2023). However, advancements in the field of genetics have unraveled responsive prostate cancer sites besides the AR (Ren, Chen, et al. 2023; Ren, Li, et al. 2023), such as poly (ADP‐ribose) polymerase, which is a target for PAPP inhibitor drugs for DNA repair abnormalities, and PD1, whose blockage can be employed in incompatible repair insufficiency (de Bono et al. 2020).

This developing area of medicine requires updated genomic sequencing approaches for patients' genomic classification and selection to enable therapeutic customization. Unfortunately, research on prostate cancer in the Asian population has not received much attention, and the majority of published data from observational and interventional studies have centered on the Western population (Zhang et al. 2023).

The lack of evidence hinders the development of specialized diagnostic and therapeutic strategies for Asian males. This study presented a thorough analysis of the epidemiology and genomics of prostate cancer in the Asian community with a particular focus on the ways to address these issues to enhance patient treatments in this community. Although it is a fact that the Asian population is heterogeneous, the focus of this review is majorly on East Asian males because these ethnic groups have comparable genetic and cultural roots (Wei et al. 2023).

### 3.3 Pancreatic Cancer

The pancreas is an approximately 6‐in.‐long, tapered, funnel‐shaped organ in the abdomen in front of the spine behind the stomach (Halbrook et al. 2023). A typical, healthy pancreas comprises centro‐acinar cells, in which a transitional area between two cells (acinar and ductal) secretes a bicarbonate solution known as hormones (Lilly et al. 2023).

Pancreatic tumors develop due to uncontrolled DNA mutations, which make the pancreatic cells expand and undergo inexorable division (Balzanelli et al. 2023). Pancreatic carcinoma is widely regarded as a lethal cancer and is among the most aggressive cancers worldwide (Luo et al. 2020; Jagadeesan et al. 2021).

Pancreatic cancers are sometimes daunting to diagnose at initial stages, and they manifest when the tumor has spread to other body areas. Pancreatic cancer, known as adenocarcinoma (PDAC), is a medical terminology referring to cancer developed in the epithelial lining of glandular tissues inside ductal glands (Elhariri et al. 2023). The pancreatic ductal adenocarcinoma (PDAC) is a cause of more than 90% of all pancreatic cancers (Nakaoka et al. 2023).

PDAC is the 10th most frequent cancer and has poor survival chances, making it the seventh most lethal cancer (Menini et al. 2021). Other cancers, such as squamous, adenosquamous, giant, and acinar cell carcinoma, are less frequent cancers of exocrine pancreatic malignancies. Currently, pancreatic cancers are still a fatal condition, and throughout the past 20 years, the prognosis has essentially stayed unaltered (Schepis et al. 2023).

The clinical outcomes of patients can be improved by clear epidemiological understanding, appropriate prevention, and early detection of disease (Luo et al. 2020). Consequently, it is essential to comprehend the epidemiological traits and growth patterns and determine the risk factors of pancreatic carcinoma in depth to design logical preventative strategies for clinical advantage.

### 3.4 Breast Cancer

According to a report by Li et al. (2024), precisely 2 million women were detected with breast carcinoma, making up 12% of all cancer patients. According to this data, breast cancer is responsible for about one in four incidences of cancer in women, demonstrating its high occurrence rate. Breast cancer is the leading cancer that affects women globally. Breast cancer has a high prevalence in comparison to other malignancies due to its high‐risk factors related to anthropometry, diet, menstruation, reproduction, delaying childbearing, and hormone consumption. Moreover, certain factors, such as hereditary susceptibility, increase the susceptibility of breast cancer in succeeding generations of high‐risk groups (Kast et al. 2023).

The treatment of breast cancer is a real challenge because the pathogenesis of BC is not homogeneous. Breast cancer has both immediate visceral and distant metastatic capacity (Nathanson et al. 2023). Endocrine receptors, endothelial growth factor receptors (EGFR), and specific proliferation markers can all manifest in certain breast cancer types, making it quite aggressive. They vary in metabolism, cell division, muscle growth, and intercellular communication. The type of breast cancer is determined by the interaction of mutations and copy number variations, whereas changes in epigenetic regulation first cause cancer to develop (Pinzaru and Tavazoie 2023).

Mostly, patients suffer from drug resistance, replacement, or therapeutic failure. The aggressiveness, diversity, metastasis, treatment resistance, relapse, and delayed diagnosis are the primary causes of a patient's high mortality. Breast tumors comprise stromal cells surrounding the cancer cells and blood cells essential for the growth and dissemination of the malignancy (Zhao, Shen, et al. 2023; Zhao, Huang, et al. 2023). As illustrated, soluble substances, suppressor immune cells, and modified extracellular matrix (ECM) all cooperate to hinder antitumor immunity while promoting metastasis. A poor prognosis for breast cancer is attributed to stromal cells with disrupted molecular processes, aberrant signaling pathways, and interactions with other TME components (Swaminathan et al. 2023).

Moreover, the ECM can alter molecular and metabolic processes. Furthermore, the molecular profile of stroma‐ECM affects the development along with the phenotype of cancer that is resistant to the treatments of cytotoxic and hormone‐based drugs (Standing et al. 2023). Correspondingly, the stroma associated with cancer (CAS) is responsible for the overexpression of gene‐encoding ECM, mitochondrial ribosome proteins, cell life cycle proteins, and matrix metalloproteinases (MMPs) during the transition from pre‐invading to invading phenotypes. Furthermore, CAS is also associated with the overexpression of immune response‐related genes (Deepak et al. 2020).

The molecular processes driving adiposity alterations in the TME encourage breast cancer development by modifying the activities of adiposities, causing the death of fat‐storing cells and establishing a non‐aggressive inflammatory response. The infected TME causes infiltration and restructuring of immune cells and promotes tumor aftermath to maintain irrevocable metastatic development (Khalaji et al. 2023). Solid tumors are distinguished by their tumor angiogenesis, a potential target for breast tumor metastasis (Marquardt et al. 2023).

Metastatic breast cancer can spread to other organs by secreting excessive amounts of proangiogenic substances, which induce atypical vascularization surrounding the tumor. Tumors have a highly disordered and leaky vascular network (Wang, Gümüş, et al. 2023; Wang, Huang, et al. 2023). The atypical vascularization develops insufficiently perfused cancers in a low‐oxygen environment TME. This environment favors severely inductive and violent tumor cells that may elude immune cells designed to destroy tumors that thrive in the hypoxic environment. Moreover, the vascular permeability factor reduces immunity by stimulating neovascularization (Choi and Jung 2023).

Additionally, irregular circulation reduces the effectiveness of radiation and chemotherapy treatments. Besides cancers, the leukocytes and stromal cells also support the growth of the tumor by producing exosome chemokines, cytokines, and proteases. More individualized therapy approaches that target the different pathophysiology of breast cancer are required to comprehend its complex biological makeup (Nail et al. 2023).

For this purpose, several approaches were introduced; one is marine sources, which hold enormous promise and can aid breast cancer therapy by discovering and developing therapeutic models. Since 1980, biotechnology advancements have opened up new fields of study into the medicinal potential of marine creatures to create innovative drugs. This creative research using state‐of‐the‐art scientific instruments has brought the potential of marine crustacean, microbial, and natural fish products as scaffolding for rational drug discovery (Harun‐Ur‐Rashid et al. 2023).

These marine chemicals can reserve novel chemical entities, new medications, and drug leads. They serve as the foundation for more than half of the medicines and approximately 80% of the chemotherapeutics authorized by the Food and Drug Administration (FDA) of the United States. Currently, marine chemicals are utilized to treat 87% of illnesses, notably cancer, by causing the death of cancer cells by concentrating on regulators of several signaling pathways or by blocking the development of tumor cells (Doghish et al. 2023).

Recent technological breakthroughs and studies on marine chemicals have enabled the formation of effective anticancer drugs that are presently being tested on animals and humans. In the present review, we discussed the developments in breast carcinoma physiology, encompassing heterogeneity, molecular and cellular physiology, and epidemiology (Swaminathan et al. 2023).

Additionally, we reviewed current research on the pathophysiology of breast cancer, such as the function of TME elements in angiogenesis, metastasis, and treatment resistance. Then, we analyzed anticancer substances derived from marine microbes, fish, and crustaceans and their interaction with anticancer medications. In our final section, we reviewed the pathophysiology of breast cancer and suggested future directions for developing marine‐based BC treatments.

### 3.5 Lung Cancer

Lung cancers are the leading cause of cancer deaths and new cases worldwide. Lung carcinoma is classified into two categories depending on the origin of the cell: small‐cell lung cancer (SCLC) and non‐small‐cell lung tumors (NSCLC) (Li et al. 2023). According to a report published by WHO, the most frequent kinds of lung cancers are adenocarcinoma (cancer of glandular cells), squamous cell carcinoma (SCC), and neuroendocrine malignancies, including small‐cell carcinoma (SCLC) and large‐cell neuroendocrine carcinoma (LCNEC) (Huang et al. 2023).

SCLC develops from poorly differentiated cells of neuroendocrine (Kulchitsky cells), leading to rapid and uncontrolled metastasis, poor response to therapy, and slow prognosis, whereas carcinoid tumors are well‐established tumors. SCLC and squamous cell malignancies, especially in men, are much more likely to be centrally situated and linked to smoking history (Wang, Gümüş, et al. 2023; Wang, Huang, et al. 2023).

Women, on the other hand, and those without a history of smoking are more prone to develop adenocarcinoma, which is characterized by peripheral onset and the presence of controllable driver aberrations in the EGFR, ALK, BRAF, and ROS1 genes. Recently, suitable individuals with these mutations have been treated with several immunetherapies such as antagonists of apoptotic protein 1 (PD‐1) and cytotoxic T‐lymphocyte‐associated protein 4 (CTLA‐4) in place of or in addition to chemotherapy (Hou et al. 2023).

In the West, smoking is responsible for 80% of lung cancer cases, and cessation has reduced the disease's incidence and mortality. In developing countries, smoking is the major causative factor of lung cancer. Still, there are other factors besides smoking, such as exposure to asbestos and gases, along with other environmental pollutants, such as arsenic, which can also cause lung cancer. A deeper and more detailed understanding of the pathophysiology and risk factors for lung cancer can aid the development of preventative strategies and reduce the rising global disease burden (Bhat et al. 2023).

According to the most recent GLOBOCAN statistics, two million ninety‐four thousand suspected cases of lung cancer were reported globally in the year 2018, earning it the highest rank of most prevalent cancer worldwide (Di Carlo et al. 2024). After breast and prostate cancer, lung cancer is expected to have one million three hundred sixty‐nine thousand cases. Thus, it ranks as the second most common kind of cancer in both genders. The age‐standardized cumulative lifetime risk of lung cancer diagnosis is 3.8% for males and 1.77% for females worldwide (Thandra et al. 2021).

The lung is more prevalent in developing countries compared to developed countries because of the increase in cigarette smoking in these countries. Lung cancer is the most frequent cancer in 37 countries, including China and several areas of Europe, the Middle East, and Asia. On the other hand, in 104 countries, men are most likely to have prostate cancer (Thandra et al. 2021).

Hungary has the highest proportion of the male population having lung cancer at a rate of 77.4 cases out of 100,000 persons. Worldwide, women's incidence rates are highest in North America, Western Europe, and Northern America (Wéber et al. 2023). The lowest prevalence is among males and women in Western, Central, and Eastern Africa (Liu et al. 2023).

The Surveillance, Epidemiology, and End Results (SEER) program of the National Cancer Institute anticipates that 229,000 newly detected lung cancer cases will be in the US, making up 12.7% of all cancer diagnoses. The highest rate of lung cancer cases globally was reported in 1992 at 69.5/100,000. Since then, the cases have dropped, primarily attributable to smoking reduction among the population. At present, the rate of lung cancer is 45.6/100,000. While the trend is reported in many Western countries, lung cancer incidence has not decreased as much in emerging countries like China and the former Soviet Union (Li et al. 2023).

In China, smoking is common in males. Most men start smoking in their mid‐20s, indicating a long‐term rise in the overall cases of lung cancer (Mohammed et al. 2023). The prevalence of lung cancer is on the rise globally due to rapid industrialization and the availability of cigarettes (Shehata et al. 2023).

## 4 Bioavailability of Nutrients

Specific nutrition management strategies are needed for some pathological states. The body's capacity to absorb and use nutrients is known as bioavailability and is influenced by various circumstances. Mushrooms make great functional meals with selenium, vitamin D2, ergothioneine, vitamin B1, iron, and more. Interestingly, the kind of mushroom influences the bioavailability of each nutrient 0.4–2.0 mg/g (dry weight) of ergothioneine, found in mushrooms (sulfur‐containing amino acid) Singh. Several researchers say ergothioneine has excellent human bioavailability (Halliwell et al. 2023).

## 5 Anticancer Substances of Mushroom Origin

**FIGURE 1:**
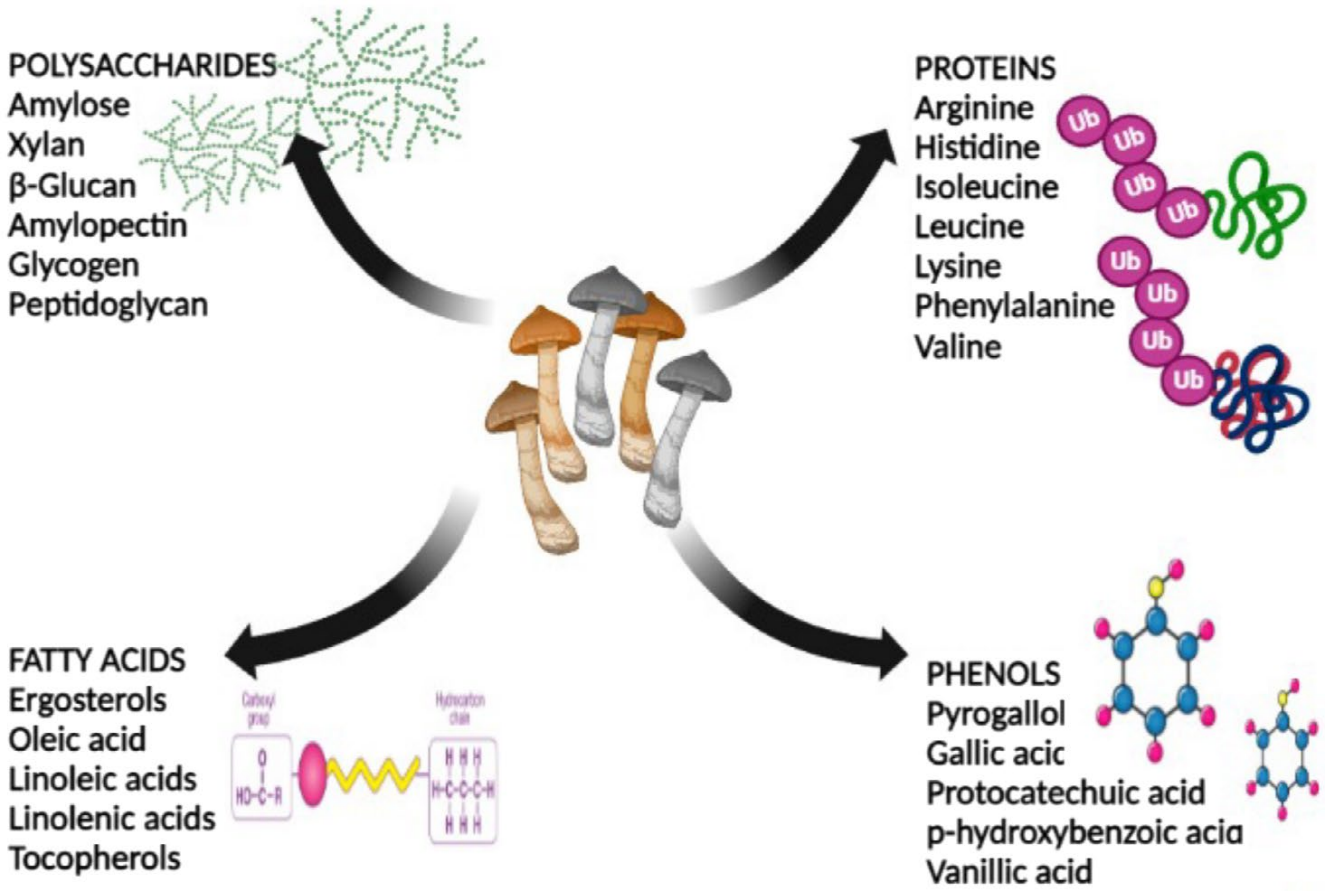
Anticancerous components of mushrooms.

Certain types of proteins, dietary fiber, polysaccharide–protein complexes, steroids, phenols, terpenoids, and other substances are among the biologically active components of medicinal mushrooms that have an anticancer effect (Figure 1). Different extracts from the fruit bodies or mycelium of *Pleurotus* mushrooms have been found to be potential anticancer nutraceuticals in several cancer cell lines and trial animals. *Pleurotus* mushrooms have anticancer properties; however, there is no good clinical proof yet (Table 1; Shreya et al. 2023).

**TABLE 1:** Anticancerous components of mushrooms.

Species	Anticancerous substances	References
Oyster mushroom (*Pleurotus ostreatus*)	Resveratrol	Nnemolisa et al. (2024)
	Cibacron blue affinity purified protein	Isanapong et al. (2024)
	Pleurotus ostreatus mycelium polysaccharides 2 (POMP2)	Meng, Jang, et al. (2023); Meng, Niu, et al. (2023)
	Pleurotus ostreatus polysaccharides‐1 (POPS‐1)	Radzki, Skrzypczak, et al. (2023); Radzki, Tutaj, et al. (2023)
	Concanavalin A	Alotaibi et al. (2023)
	Pleuran	Hassan et al. (2023)
	Ergosta‐7,22‐dienol	Tejedor‐Calvo et al. (2023)
	α‐tocopherol	Zhong et al. (2024)
	Ergosta‐5,7‐dienol	Sommer et al. (2024)
	Ergosta‐7‐enol	Pandey et al. (2024)
	Ergosterol	Kaur et al. (2023)
Button mushroom (*Agaricus bisporus*)	Gallic acid	Mehrdel et al. (2023)
	Protocatechuic acid	Bai et al. (2023)
	Catechins	Baris et al. (2023)
	Caffeic acid	Zhou et al. (2023)
	Ferulic acid	Radzki, Skrzypczak, et al. (2023); Radzki, Tutaj, et al. (2023)
	Myricetin	Mehrdel et al. (2023)
	Polysaccharides	Liu et al. (2024)
Straw mushroom (*Volvariella volvacea*)	Quercetin	Koffi et al. (2023); Cheng et al. (2024);
	Catechin	Tapnio (2023)
	B‐Glucan	See Toh et al. (2023)
	Lovastatin	Şöhretoğlu and Kuruüzüm‐Uz (2023)
	Eritadenine	Rauf et al. (2023)
	Lentinan	Kumar et al. (2021)
	Peptidoglycan	Sarkar and Archana (2022)
Hen‐of‐the‐Woods or Maitake mushroom (*Grifola frondosa*)	Proteoglucan	Ray et al. (2024); Zhu et al. (2022)
	Galactose	
	Dxylose	
	d‐fucose, d‐mannose, l‐arabinose	
	uronic acid	
	d‐mannose	
	l‐arabinose	
Shiitake mushroom (*Lentinula edodes*)	Polysaccharide	Ray et al. (2024); Yamaguchi et al. (2011)
	Lentinan	
	Lysozyme	
	Beta‐Glucan	
	Glucan	
*Monascus purpureus*	Indole compounds	Ray et al. (2024); Zhu et al. (2019)
	Ergosterol	
	Ergosterol peroxide	
	Hexestrol	
	Cholecalciferol	
	α‐d‐glucopyranose	
Turkey tail mushroom (*Coriolus versicolor*)	Proteins	Ray et al. (2024); Cruz et al. (2016)
	Fatty acids	
	Polysaccharides	
	Polysaccharopeptides	
	Glucans	
	Amino acids, and vitamins	
Umbrella Polypore (*Polyporus umbellatus*)	Amino acids and analogues	Ray et al. (2024); Zhao et al. (2016); He et al. (2022)
	Flavonoids	
	Nucleosides and analogues	
	Phenols and steroids	

In one investigation, human prostate cancer cells were treated with a water‐soluble extract from fresh *P. ostreatus*, which caused significant cytotoxicity and apoptosis (Akyüz et al. 2023). *P. ostreatus* hot water extract also reserved the growth of human breast cancer cells. Some colon and breast cancer cells have been studied to see how *P. ostreatus* methanol extract affects them. A flow cytometry study found that *P. ostreatus* stops the cell cycle in MCF‐7 and HT‐29 cells at the G0/G1 phase, which prevents the cells from proliferating (Krishnakumar et al. 2023).

Other *Pleurotus* mushrooms effects on breast and colon cancer are equally encouraging. Colon cancer cell lines (HT29, HCT116) were less likely to attach when polysaccharide extracts from *Pleurotus pulmonarius* mycelium and fruit bodies were present. This directly impedes the growth and spread of the disease. The polysaccharides from a hot water extract of *P. geesteranus* showed significant cytotoxicity in the human breast cancer cell line MCF‐7 (Gafforov et al. 2023).

It has also been found that *Pleurotus* mushrooms contain anticancer qualities that are considered in experimental animals, mainly rats and mice. In mice with colon cancer, which was induced experimentally, treatment with *P. ostreatus* powder decreased, suggesting that it may be useful as a treatment for inflammation‐related colon carcinogenesis (Ren, Chen, et al. 2023; Ren, Li, et al. 2023).

The culture broth in nature is the extract from *Pleurotus ostreatus* fruiting bodies on female Swiss albino mice that had been injected with the Ehrlich ascitic tumor. When administered intraperitoneally to mice, every chemical tested reduced the growth of tumors at rates close to 70%. Female Swiss mice that had been infected with the Ehrlich ascitic tumor likewise showed a more significant decrease in the number of neoplastic cells when the polysaccharide fractions of *Lentinus sajor‐caju* were administered intraperitoneally (Gafforov et al. 2023).

When given intraperitoneally, a new lectin isolated from *P. citrinopileatus* significantly slowed the development of tumors and exhibited strong anticancer efficacy in mice harboring sarcoma 180. It has also been demonstrated in experimental mice that *P. florida* possesses antioxidant and anticancer properties. *P. pulmonarius* extracts added to the diet of CBA mice with chemically generated hepatocellular carcinoma prevented the course of carcinogenesis (Inci et al. 2023).

In most cases, it was discovered that the polysaccharide portion of *Pleurotus* mushrooms is the primary factor behind their anticancer efficacy. One of the constituents of polysaccharide beta‐glucans, a significant component of *Pleurotus* species, is pleuran. This ingredient significantly reduces cancer risk and boosts immunity (Nandi et al. 2023).

While there are other anticancer agents, one is a 9‐kDa ribonuclease (RNase) from *P. ostreatus* fresh fruit bodies, which exhibited antiproliferative action against leukemia cells and an N‐terminal sequence. Once more, *P. djamor* was used to purify a 15‐kDa RNase with antiproliferative effects on tumor cells (HepG2 and MCF7). Ngai and Ng identified a 12‐kDa RNase from the more active fresh fruit bodies of the *Lentinus sajor‐caju* mushroom toward poly‐U and significantly less active against poly‐A, poly‐G, and poly‐C (Gafforov et al. 2023).

The RNase decreased the possibility of the tumor cells HepG2 (hepatocellular carcinoma) and L1210 (leukemia). Fresh fruit bodies of *P. citrinopileatus* were used to extract a useful protein (PCP‐3A), which prevented the growth of the U937 human leukemia cell. Flow cytometry research showed that PCP‐3A can prevent U937 cells from proliferating by causing an S phase arrest and apoptosis induction.

Identified a new hemolysin from *P. nebrodensis* , and it demonstrated potent cytotoxicity contrary to HeLa, Lu‐04, Bre‐04, and HepG2 cancer cells, as well as murine fibroblasts and breast cancer cells (cervical cancer). L929 and HeLa cells were likewise subjected to apoptosis by this hemolysin, *Pleurotus ostreatus* mycelia water‐soluble proteoglycan fractions, as immunomodulators and anticancer agents (Krishnakumar et al. 2023).

A mouse model bearing Sarcoma‐180 was examined for immunomodulatory and anticancer effects in vitro and in vivo. The number of tumor cells decreased, and cell cycle analysis showed that the majority of tumor cells were found to be in the pre‐G0/G1 phase stopped when Sarcoma‐180‐bearing animals got proteoglycan injections in vivo (Bhagarathi et al. 2023).

Additionally, these proteoglycans increased the cytotoxicity of natural killer cells in mice and promoted the production of nitric oxide by macrophages. Mammalian DNA topoisomerase I was inhibited by a chloroform extract of *P. eryngii* (Gafforov et al. 2023). The inhibiting substance, ubiquinone‐9, was isolated and identified as the culprit. It did not affect normal fibroblast cells, but it did affect human leukemia cells. Additionally, it was proven that ubiquinone‐9 can induce apoptosis. The inhibiting substance, ubiquinone‐9, was isolated and identified (Tuyet and Kim 2023).

### 5.1 Polysaccharides and Polysaccharide–Protein Complexes

Macromycetes fruiting bodies, mycelial mass, and culture broth included biologically active polysaccharides. Mushroom polysaccharides exhibit immune cell‐mediated anticancer action in addition to preventing carcinogenesis and metastasis. The majority of polysaccharides with anticancer activity are glucans, which have side branches with β‐(1 → 3) bonds and β‐(1 → 3) bonds in the main chain. Side branches are necessary for the incidence of biological activity (Krishnakumar et al. 2023).

Additionally, it was shown that polysaccharides with high molecular weight are more efficient than polysaccharides with low molecular weight Even though it is commonly known that providing cancer patients with mushroom polysaccharides can be an alternate treatment option, it makes sense to combine these substances with more conventional forms of therapy, including surgery, chemotherapy, and radiotherapy. The adverse effects of traditional cancer therapies can be lessened when polysaccharides, polysaccharide–protein complexes, and mushroom extracts are used together (Rokos et al. 2023).

It is essential to highlight that clinical studies for medications derived from mushrooms are sufficiently costly and time‐consuming. As a result, several studies on the anticancer properties of medicinal mushrooms have been conducted in vivo and in vitro. But as far as we are aware, not many polysaccharides or polysaccharide–protein complexes have been through successful clinical trials (Hu et al. 2023).

### 5.2 Lentinan

The primary chain of lentinan, which has a molecular weight of 500 kDa, is made up of glucose and is coupled to side chains by β‐(1 → 3) and β‐(1 → 6) bonds. Lentinan attaches to the surface of lymphocytes or specific proteins in blood serum, activating macrophages, T‐killer cells, and other effector cells (Swallah et al. 2023).

This increases the production of antibodies, IL‐1, IL‐2, and IFN‐. Sarcoma‐180 in mice implanted subcutaneously grows inactively in the presence of lentinan. In clinical trials, tegafur was administered to patients with stomach cancer. The survival rates increased at the same rate (2.9%) in the first and second years but not in the third year. Survival rates increased by 19.5%, 10.4%, and 6.5% in patients who got tegafur and lentinan concurrently for 1, 2, and 3 years (1 mg twice weekly or 2 mg once weekly managed intravenously) (Zheng et al. 2023).

In cases of rectal cancer that was developing and relapsing, similar results were seen. Similar results did not occur when lentinan was combined with the anticancer drugs mitomycin C and 5‐fluorouracil. Lung, breast, and stomach cancer treatments were made more effective by combining lentinan with radiation and surgery. Rare occurrences of lentinan toxicity and adverse responses have been reported (Dan et al. 2023).

### 5.3 Schizophyllan

Lentinan and schizophyllan have similar structures and anticancer effects. Schizophyllan, a (1 → 3)‐β‐glucan with (1 → 6)‐β‐glucopyranoside branching, is linked to every third or fourth residue. Schizophyllan helps the tumor‐bearing host's cellular immunity by acting as a macrophage activator, a T‐cell adjuvant, and a promoter of cytokine production (Stoica et al. 2023).

Schizophyllan has little anticancer effect when provided subcutaneously, but when delivered intravenously or intraperitoneally, it inhibits solid Sarcoma 180 and extends mouse life. Mice with Sarcoma 37, Yoshida sarcoma, or Ehrlich's carcinoma do not affect their survival. Clinical trials with schizophyllan combined with conventional treatment increased the median survival of 367 patients with recurring and incurable stomach cancer considerably. Recent research has shown that schizophrenia increases general survival in people with head and neck cancer (Peters et al. 2023).

Schizophyllan significantly extended stage II cervical cancer patients overall survival in a randomized trial when radiation was added, but not for patients at stage III. Schizophyllan, approved for clinical usage in Japan, is commercially produced by several pharmaceutical companies in Japan (Paul et al. 2023).

*T. versicolor* , a basidiomycete, produced two distinct polysaccharide fractions with anticancer characteristics. Using hot water extraction, the Japanese business Kureha Kagaku Kogyo K.K. produced PSK (krestin), a D‐glucan–protein complex (Tang et al. 2023). The krestin anticancer activity mechanism includes the following processes: restoration from immunosuppression brought on by humoral factors like TGF‐ or as a result of surgery and chemotherapy; initiation of antitumor immune reactions, including dendritic cell maturation, and improvement of the antitumor consequence of chemotherapy by inducing apoptosis and preventing metastasis (Ma et al. 2023).

### 5.4 Proteins

The most researched bioactive element of mushrooms is polysaccharides. But another important component of the functional elements in mushrooms is bioactive proteins, which are also gaining popularity because of their potential as drugs and the ability to create protein engineering with specific capabilities (Fernandes et al. 2023).

Mushroom proteins with anticancer properties may be split into immunomodulating proteins and proteins with direct antiproliferative effects on cancer cells. Both of these action methods may be found in the class of lectins. The most prevalent cancer cell lines utilized to test the antiproliferative effect of mushroom proteins in vitro were Hep G2 and MCF‐7 (Akyüz et al. 2023). Pathways of different mushrooms on cancers and their mechanisms are shown in Table 2.

**TABLE 2:** Pathways of different mushrooms on cancers and their mechanisms.

Species	Types of cancer	Experimental model	Solvent/Fraction	Mechanism/Results	References
*Pleurotus ostreatus*	Breast cancer	In vitro (MDA‐MB‐231 cells and MCF‐7)	Methanol extract	The extract had an antiproliferative impact that caused the cell cycle to stop in the G0/G1 phase. Additionally, it boosted p21 (a cyclin‐dependent kinase inhibitor) and p53 (a tumor suppressor) levels while lowering Rb phosphorylation activity to cause p53‐independent apoptosis	Bhekti Rahimah et al. (2023)
*Pleurotus ostreatus*	Breast cancer	In vitro (MCF‐7)	Methanol extract	The component showed antiproliferative effects on cancer cells	Zhao et al. (2024)
*Pleurotus ostreatus*	Colon cancer	In vitro (COLO‐205 cells)	Water extract	The cell cycle was arrested at the Go/G1 phase. Apoptosis was induced as a result of increased production of cyclin‐dependent kinases (CKIs), p16, and p21) BAX, proapoptotic genes (caspase‐3, caspase‐9), and decreased expression of anti‐apoptotic genes in the extract, which is what gave rise to the anticancer action (BCL2)	Pandey et al. (2023)
*Pleurotus ostreatus*	Colon cancer	In vitro (HT‐29 and HCT‐116 cells)	Methanol extract	The extract had an antiproliferative impact that caused the cell cycle to stop in the G0/G1 phase. Additionally, it boosted levels of p21 (a cyclin‐dependent kinase inhibitor) and p53 (a tumor suppressor) while reducing Rb phosphorylation activity to trigger p53‐independent apoptosis	Tülüce et al. (2023)
*Pleurotus ostreatus*	Colon cancer	In vivo – mice model	Ethanol extract	Preventing the formation of F4/80, Ki‐67, COX‐2, and cyclin D1 in mice was connected with the anticancer mode of action, which involved the prevention of inflammatory mediators tumorigenesis	Khinsar et al. (2023)
*Pleurotus ostreatus*	Colorectal cancer	In vitro (Colo‐205 cells)	Methanol extract	The component showed antiproliferative effects on cancer cells	Shreya et al. (2023)
*Pleurotus ostreatus*	Leukemia	In vitro (Jurkat and KG1 cells)	Water Extract	Due to increased BAX gene expression, the fraction reduced migration, cell proliferation, and expression of matrix metallopeptidase‐9, and it promoted apoptosis	Lesmana et al. (2023)
*Pleurotus ostreatus*	Leukemia benzene‐induced	In vivo – rat model	Water extract	Leukopoiesis was accelerated by the extract, which also showed phagocytic qualities	Vishwakarma et al. (2023)
*Pleurotus ostreatus*	Melanoma cancer	In vivo – mice model (B‐16 cells)	Water extract	Antitumor activity was based on immune system stimulation	Shamtsyan et al. (2023)
*Pleurotus ostreatus*	Prostate cancer	In vitro (PC‐3 cells)	Water extract	The extract caused cancer cells to undergo apoptosis	Pandey et al. (2023)
*Pleurotus ostreatus*	Erythroleukemia	In vitro (KG‐1 cells)	Methanol extract plus doxorubicin hydrochloride	Combining the fraction with doxorubicin hydrochloride induced apoptosis and a considerable boost in the anti‐cancer effects	Shreya et al. (2023)
*Pleurotus ostreatus*	Breast cancer	(MCF‐7, MDA‐MB‐231)		*P. ostreatus* stops breast and colon cancer cells from proliferating	Al‐Rajhi and Ghany (2023)
	Colon cancer	(HT‐29, HCT‐116) cells			
*Pleurotus ostreatus*	Prostate cancer	PC‐3 cell		The beginning of apoptosis	Matkovits et al. (2024)
*Pleurotus ostreatus*	Leukemia	L1210 cell		RNase activity controls the expression of proliferative genes	Yan et al. (2023)
*Pleurotus ostreatus*	Breast cancer and colon cancer	(MCF‐7, MDA‐MB‐231 cells) (HT‐29, HCT‐116 cells)		MCF‐7 and HT‐29 up‐regulation of p53 and p21, cells exhibit cell cycle arrest and down‐regulation of Rb phosphorylation	Zhang et al. (2025)
*Agaricus bisporus*	Prostate cancer	In vitro (PC3 cells)	Ethanol extract	The extracts reduced cell growth by modulating IL‐8 production and lowering VEGF levels. Additionally, mushroom extracts reduced NF‐B and nuclear activity	Akyüz et al. (2023)
*Agaricus bisporus*	Breast cancer		Aqueous extract	Suppress MCF‐7aro cell growth and aromatase activity, which suggests a decrease in estrogen levels (breast cancer cell lines)	Zhao et al. (2024)
*Agaricus bisporus*	Immunomodulation		Alpha‐glucans	The consumption of fruit juice fortified with 5 g of glucans per day reduced the generation of TNFa caused by lipopolysaccharide by 69%. There were no impacts on IL‐1b and IL‐6, whereas IL‐12 and IL‐10 production were reduced (in vivo). On the other hand, in an in vitro mouse model, alpha glucans have been demonstrated to increase immunological response	Bindhu et al. (2023)
*Agaricus bisporus*	Leukemia			HL‐60 leukemia cells and other leukemia human cell lines' ability to increase is inhibited by inducing apoptosis	Lesmana et al. (2023)

### 5.5 Lectins

Without affecting the covalent structure of any known glycoside ligand, lectins are multivalent proteins that can accurately detect and attach themselves reversibly to glycoconjugates carbohydrate moiety (Nagao et al. 2023). Lectins provide various biological roles, including helping to mobilize and transport sugars, recognize cells, control development, and differentiate cells. They also help parasitic fungi infiltrate their host's bodies and bond with higher plants during mycorrhization. Of all the mushroom proteins, lectins have likely undergone the most research. Notably, the analysis of membrane glycoconjugate alterations and cancer development, the categorization of tumor and mutant cells, diagnostics, and prodrug creation are all done with the assistance of mushroom lectins. Certain lectins can agglutinate cells and have mitogenic action toward specific cell types, such as immune cells, and antiproliferative action versus malignant cells (Sousa et al. 2023).

The ricin B‐like lectin from *Clitocybe nebularis* is one example of a lectin that has direct cytotoxicity against cancer cells, which can identify carbohydrates that determine a person's blood type and has antiproliferative action only against human leukemic T cells. HeLa cells are cytotoxic to a lectin derived from the maturing bodies of *G. frondosa* that belong to the same glycoprotein class. Cancer cells are less likely to increase and become mitogenic when exposed to the lectins from *Polyporus adusta* and *Ganoderma carpense*, respectively (Hammond et al. 2023).

### 5.6 Cytotoxic Terpenoids

Terpenes are bonded to an oxygen‐containing group to form a family of hydrocarbons known as terpenoids. Terpene, conversely, is any member of the group of hydrocarbons composed of two or more interconnected units of isoprene (C_5_H_8_). Numerous studies focus on the cytotoxic properties of the triterpenoids found in the basidiomycete *Ganoderma lucidum* (containing six isoprene units). More than 150 triterpenoids have been found in the fruiting bodies of the *Ganoderma* genus. The polyoxygenated ganoderic triterpene acids T, V, W, X, Y, and Z extracted from *G. lucidum* exhibited cytotoxic action against the HTC cell line in vitro, as reported by Singh et al. (2023).

According to research, the triterpenoid fraction from *G. lucidum* fruiting bodies includes ganoderic acid F. It has anticancer and antimetastatic properties by preventing angiogenesis, a process that tumors bring on. However, it had no similar effects on normal hepatocytes. It has previously been demonstrated that oxidative stress plays a role in the cytotoxicity of this fraction of tumor cells (Chen, Wang, et al. 2023; Chen, Lei, et al. 2023).

Three triterpene aldehydes called lucialdehydes A, B, and C have lanostane skeletons generated from *G. lucidum* fruiting bodies. The highest level of cytotoxicity against the examined cells was shown by lucialdehyde C. It was demonstrated that *G. lucidum* intracellular triterpenoids can occur mostly in the latter stages of submerged fermentation, differ in composition, and are toxic to K562 cells. The development of semi‐synthetic analogues with specific properties is a viable strategy since mushroom terpenoids have the potential to be deadly for normal cells and cancer cells. Thus, it was discovered in 1963 that *Omphalotus guepiniiformis* had a strong anticancer activity linked to the poisonous chemical lampterol due to screening from roughly 600 mushrooms (Chen, Wang, et al. 2023; Chen, Lei, et al. 2023).

*Omphalotus olearius* may also produce the cytotoxic tricyclic sesquiterpene illudin S (*lampterol*), which has three isoprene units. Illudin S is thought to be activated by glutathione. When the active form can covalently link to DNA, DNA replication stops, and cell death occurs in interphase G1‐S. Toxic in nature, illudin S is not suitable for usage in therapeutic settings. As a result, a semi‐synthetic illudin analogue called HMAF was used in clinical studies since it had a better therapeutic profile and less toxicity (Casimir et al. 2023).

In vitro, sensitivity was consistent with in vivo anticancer activity. HMAF was incredibly effective in human tumor xenograft models, such as MX‐1, MV522, and HT‐29, but not in B16 or P388. Consequently, after receiving intravenous treatment of HMAF at dosages of 3–7.5 mg/kg daily for 5 days, full remission was seen in 29 of 30 rats with MX‐1 tumors. Rats that received intraperitoneal injections of MV522 or HT‐29 for 5 days at dosages ranging from 3.75 to 7.5 mg/kg showed a significant reduction in tumor size (Gray et al. 2023). Furthermore, full regressions were observed in several animals receiving HT‐29 and MV522 treatments. Phase I of the HMAF clinical trial was satisfactorily completed. However, phase II demonstrated no efficacy, and further research has been discontinued.

### 5.7 Phenols, Dietary Fiber, and Steroids

The glycosylated form of ergosterol peroxide present in the *Cordyceps sinensis* methanol extract hindered the development of the tumor cell lines. *Sarcodon imbricatus* contains an anticancer sterol that inhibited the proliferation of HT29 cancer cells but not of WI38 normal human fibroblasts (Nandi et al. 2023).

According to research on anticancer mechanisms, the cyclin‐dependent kinase inhibitor 1A is expressed when the sterol mentioned above triggers cell cycle arrest and death in HT29 cells. Phenols can directly kill cancer cells and have an anticancer effect as a preventative agent with antioxidant characteristics. It was proposed that the β‐lactam and its N‐substituent may be the cause of the high cytotoxicity of hericenone B. Chitin, homo‐, and heteropolysaccharides are large molecular weight compounds found in mushroom cell walls that cannot be absorbed or digested by the human gut but can absorb cancer‐causing agents (Massacci et al. 2023).

The quantitative and qualitative makeup of the fungal mycelia is influenced by several cultural factors, particularly the substrate. Synthetic, readily available media can suppress the production of several critical secondary metabolites. The choice of the growth medium is one of the crucial steps in this process for getting the physiologically active components of the fungal mycelia. The most desirable materials can be natural substrates or agricultural waste (Diamantopoulou and Papanikolaou 2023).

## 6 Proposed Mechanism of Anticancer Through Mushrooms

**FIGURE 2:**
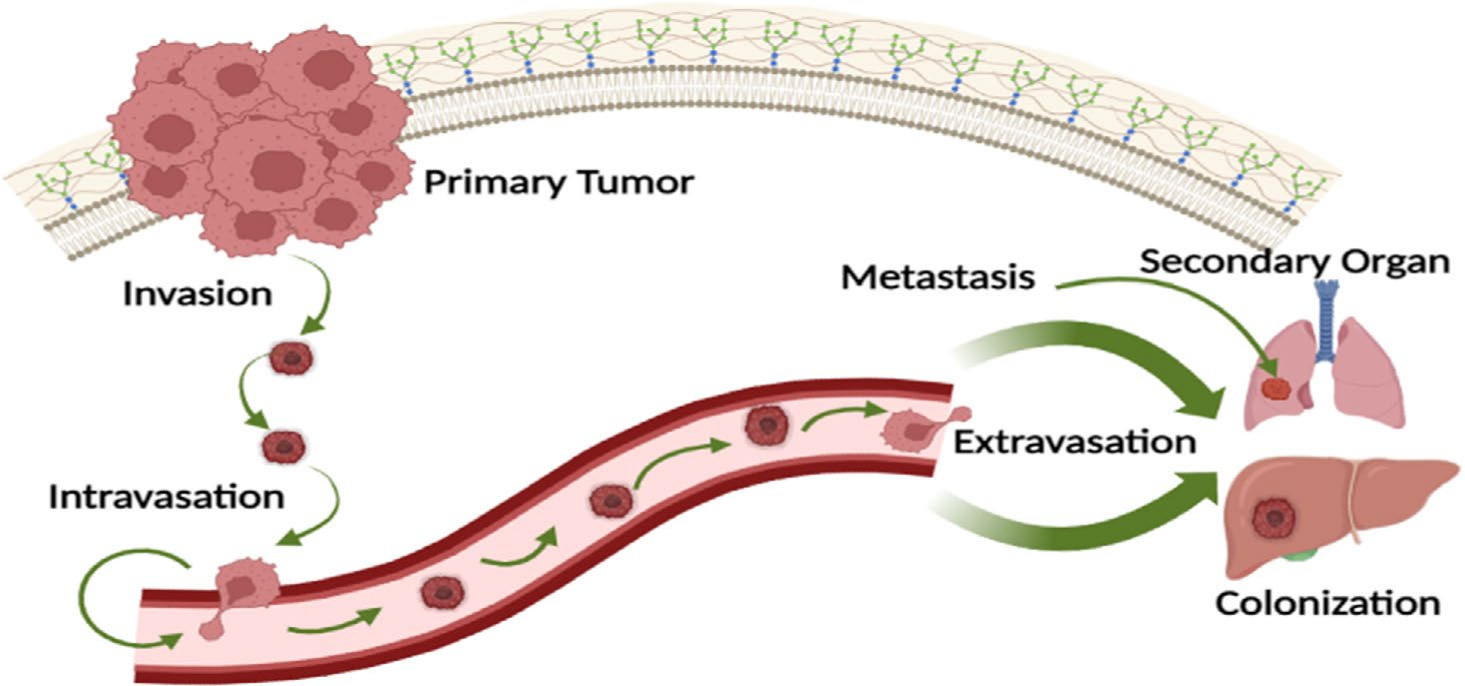
Effect of bioactive components derived from the mushroom on the cancer cell.

The effect of bioactive components derived from the mushroom on the cancer cell is presented in Figure 2. The latest research analysis conducted over mushrooms, as well as their essence, has recognized actions associating host‐conciliated immuno‐modulatory reactions through triggering of both the adaptive and the natural immune passageways, with indications to create barriers for growth of the tumor with the aid of antiproliferative effects, also with the initiation of apoptosis in cancer cells of the human body (Yang et al. 2023).

Polysaccharides from edible mushrooms, typically in the beta‐glucan group, reduce the growing tumor cells by invigorating immunity. A few of the more productive components in mushrooms are 1,3‐b‐glucans and 1,6‐branched, identified at sequence‐identification sense organs on resistant cells like granulocytes, dendritic cells, and monocytes (Guo et al. 2023).

Beta‐glucans existing in mushrooms are also familiar for their exerting immuno‐modulatory effects by activating macrophages, the stability of T‐ helper cells, which are TH1 and TH2, within distinct communities also successive results on the natural killer cells NK, together with the making of cytokine (Murphy et al. 2023). Additionally, with ‘beta‐glucan’, poly‐saccharides with more carbohydrate elements, for example, ‘alpha‐glucans’, have also been involved, while the second research with antitherapeutic mushrooms carrying ‘1,4‐glucan’ has not revealed that a similar feature recommending that splitting these ‘beta‐glucans’ can give the precision toward the tying such components to defense mechanism cell (Hasan and Abdulhadi 2023).

While most of such systems have been resolved in vivo or in vitro animal research findings, a part of new data has also given proof for these kinds of immuno‐modulatory effects (IgM, effects of IgG, neutrophil, activity of NK cell, leukocyte count) in human from oral consumption of dietary poly‐saccharides from some of the types of this food, that furthermore build up the information (Yu et al. 2023).

Antiproliferative or apoptotic reactions on cell lines and carcinomas are processes collaborated by several mushrooms and extracts in investigations for the role of anticancer. The effect of proteo‐glycan mushroom fragments as an antitumor links the increase of the number of cells of NK as well as the vitalization of non‐deductive ‘NO’ fusion gene expression that is followed further by formation of NO in macrophage through activating the NF‐kappaB and transcriptions aspect. NK cell activation is probably via interleukin and interferon‐gamma‐mediated paths (Şebin et al. 2023).

Moreover, with the effects of apoptosis and antiproliferation, the outlined antiviral/microbial and anti‐inflammatory effects may also take part in the anticarcinogenic response of mushrooms along with the extracts, even though these straight relations have not been organized until now. As stated earlier in vivo or in vitro animal research investigations, the majority of these operations have been determined, polysaccharides of mushrooms are starting to be assessed as an adjuvant cancer treatment component next to common cancer care (Narayanan et al. 2023), specifically with patients who have breast cancer with estrogen‐receptor‐positive tumor in which the extracted material of these foods is appeared for suppressing the aromatase action (Gong et al. 2023) and consecutive decrease of estrogen levels. Although the effective role and fundamental procedures of mushroom‐polysaccharides in healthcare issues are assessed more broadly, bio‐active proteins like fungal immunomodulatory, ribonucleases, lectins, ribosome‐inactivating proteins, and some of the remaining proteins have been found to possess the protection from viral infections, antitumor, and immuno‐modulatory work. Moreover, agaritine and ergosterol of the *Agaricus* mushroom class are studied by researchers to hinder the proliferating leukemia‐causing cells with no effect on the usual “lymphatic” cell, and this role was different from beta‐glucan (Jinfeng et al. 2023).

## 7 Conclusions

Most research on anticancer compounds derived from mushrooms is focused on polysaccharides, polysaccharide–protein complexes, lectins, and terpenoids. The D‐ and MD‐fractions of the polysaccharide–peptide complex (PSK) from *Lentinula edodes* (shiitake mushroom), lentinan, *Trametes versicolor*, *Grifola frondosa* (polysaccharopeptide), and schizophyllan from *Schizophyllum commune* (splitgill mushroom), as well as compounds related to hand, foot, and mouth disease (HFMD) from *Lotus japonicus*, are the only substances that have completed the initial stages of clinical studies.

A class of proteins known as mushroom lectins can have direct cytotoxic and immunomodulating effects on cancer cells. Researchers may study the association between changes in membrane glucoconjugates and cancer development, the classification of cancerous and mutant cells, diagnosis, and the synthesis of prodrugs due to lectins' ability to bind to glucoconjugates selectively. Rarely discussed anticancer mushroom proteins include ribosome‐inactivating proteins, enzyme laccase, and hemolysin proteins, which display direct cytotoxic effects in vitro.

## Author Contributions

**Ali Ikram:** methodology (equal), writing – original draft (equal). **Nasir A. Ibrahim:** data curation (equal), supervision (equal). **Muhammad Tayyab Arshad:** data curation (equal), writing – review and editing (equal). **Abroo Fatima:** supervision (equal), visualization (equal). **Ali Asghar Taseer:** conceptualization (equal), resources (equal). **Mahreen Faqeer Hussain:** conceptualization (equal), formal analysis (equal). **Zunair Abdullah:** supervision (equal), validation (equal). **Nosiba S. Basher:** conceptualization (equal), data curation (equal), resources (equal). **Muhammed Adem Abdullahi:** project administration (equal), writing – original draft (equal). **Ammar AL‐Farga:** data curation (equal), project administration (equal). **Mohammed Ali Al‐Duais:** data curation (equal), visualization (equal).

## Disclosure

Institutional Review Board Statement: This study did not involve humans or animals.

## Consent

The authors have nothing to report.

## Conflicts of Interest

The authors declare no conflicts of interest.

## Data Availability

The data that support the findings of this study are available on request from the corresponding author. The data are not publicly available due to privacy or ethical restrictions.
